# Stochasticity, periodicity and localized light structures in partially mode-locked fibre lasers

**DOI:** 10.1038/ncomms8004

**Published:** 2015-05-07

**Authors:** D. V. Churkin, S. Sugavanam, N. Tarasov, S. Khorev, S. V. Smirnov, S. M. Kobtsev, S. K. Turitsyn

**Affiliations:** 1 Aston Institute of Photonic Technologies, Aston University, Birmingham B4 7ET, UK; 2 Novosibirsk State University, 630090 Novosibirsk, Russia; 3 Institute of Automation and Electrometry SB RAS, 1 Ac. Koptyug Avenue, 630090 Novosibirsk, Russia; 4 Institute of Computational Technologies SB RAS, 630090 Novosibirsk, Russia; 5 Zecotek Photonics, Inc., 1120-21331 Gordon Way, Richmond, British Columbia, Canada BC V6W 1J9

## Abstract

Physical systems with co-existence and interplay of processes featuring distinct spatio-temporal scales are found in various research areas ranging from studies of brain activity to astrophysics. The complexity of such systems makes their theoretical and experimental analysis technically and conceptually challenging. Here, we discovered that while radiation of partially mode-locked fibre lasers is stochastic and intermittent on a short time scale, it exhibits non-trivial periodicity and long-scale correlations over slow evolution from one round-trip to another. A new technique for evolution mapping of intensity autocorrelation function has enabled us to reveal a variety of localized spatio-temporal structures and to experimentally study their symbiotic co-existence with stochastic radiation. Real-time characterization of dynamical spatio-temporal regimes of laser operation is set to bring new insights into rich underlying nonlinear physics of practical active- and passive-cavity photonic systems.

## Abstract (web summary)

The interplay of processes with different spatio-temporal scales is present in many physical systems but its study is challenging. Here, Churkin *et al*. study the dynamics of a partially mode-locked laser where stochastic and periodic processes interplay by mapping the evolution of the intensity autocorrelation function.

## Graphical abstract

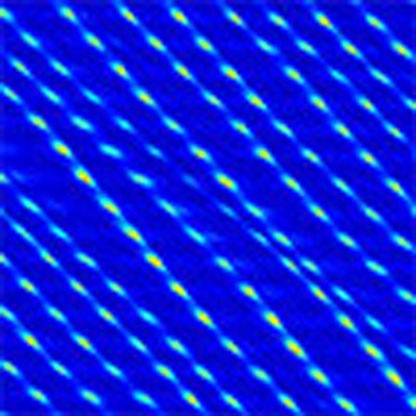

Understanding the fundamental physics behind pulse generation in nonlinear fibre resonators is critically important for the development of high-energy pulsed lasers. In conventional mode-locked fibre lasers, the pulse energy is inversely proportional to the repetition rate, offering a straightforward way to boost the energy of generated pulses by elongation of the laser cavity^1^,^2^,^3^.

However, long fibre laser systems are known for their complex behaviour, whereby phase and amplitude stochasticity arising from nonlinear interaction, scattering, amplifier noise and other effects result in a loss of coherence. As the laser cavity is elongated, fully coherent lasing operation tends to grow more and more unstable, eventually developing into stochastic generation with low coherence^3^,^4^,^5^ and noise-like pulses^6^,^7^. Nonlinear physics of long mode-locked lasers is a fast-growing area: various dissipative structures are generated under certain conditions, such as soliton rains^8^,^9^ and molecules^10^, dark solitons^11^. More complex nonlinear processes, such as soliton explosions^12^,^13^, rogue wave generation^14^,^15^,^16^,^17^ and even optical turbulence^18^,^19^,^20^, are a popular area of focus for researchers.

These highly nonlinear regimes, which are often of a dynamic, non-stationary nature, are usually shunned by engineers because of their intractability and limited practical usability compared with the standard mode-locking regimes. However, modern fibre lasers push the limits of performance, which means it is hardly possible to ignore this complexity as nonlinear intra-cavity effects play an important role in system design and limit the maximum energy that could be achieved in master oscillators^21^,^22^. Understanding and mastering the variety of nonlinear generation regimes may lead to the development of new laser types with new features and better performance, similar to optical communications where the regenerative nonlinearity helps to design systems with capacity that is higher than the corresponding linear Shannon limit^23^.

It is a challenging task to experimentally identify different partially mode-locked regimes and gain insights into the complex intra-cavity dynamics of radiation. Indeed, experimental characterization techniques are mostly designed for analysing temporal dynamics of conventional stationary mode-locking regimes; autocorrelation or FROG-based techniques^24^ work well in the case of a stable pulse train of identical pulses. For stochastic radiation that exhibits dynamic behaviour, all differences between pulses resulting from noise will be cancelled out in measurements averaged over many of them. Single-shot techniques^25^ and direct real-time intensity measurements are required to reveal the internal stochastic structure of individual pulses.

In the present study, we discovered non-trivial long-scale internal periodicity in stochastic generation of partially mode-locked fibre lasers operating in dynamic spatio-temporal regimes. By introducing an intensity autocorrelation function evolution mapping technique, we reveal constituents of thought-to-be stochastic radiation and observe quasi-stationary localized dark and bright structures and their interaction with the stochastic pulses and inter-pulse background. This technique has emerged as a new practical tool for the experimental study of underlying nonlinear physics in fibre lasers operating in spatio-temporal regimes that are neither coherent (as in traditional mode-locked systems) nor completely stochastic.

## Results

### Dynamical spatio-temporal regimes

**Figure 1:**
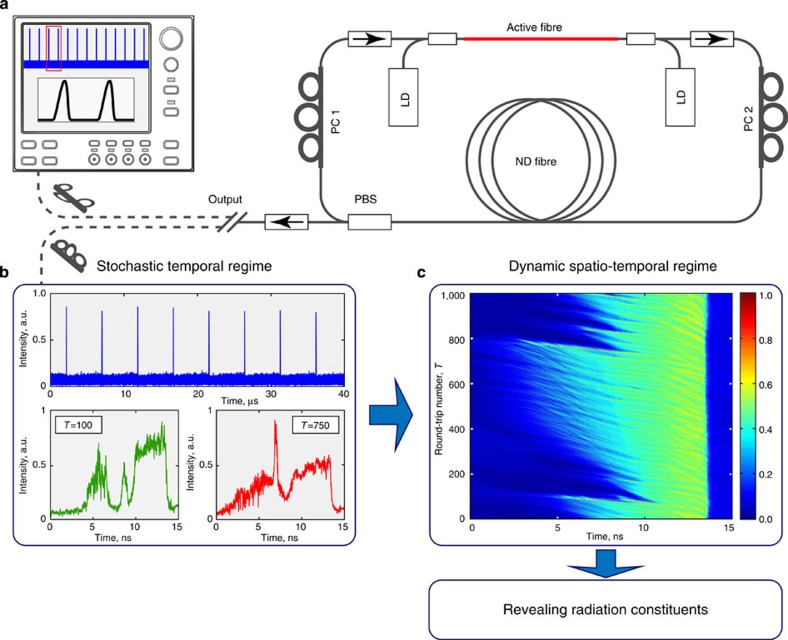
Dynamic spatio-temporal regime of partially mode-locked fibre laser. (**a**) The laser design and (**b**) its intensity dynamics, *I(t)*. The temporal shape of the noise-like pulse exhibits substantial variation while evolving over round-trips showing the stochastic nature of the generation regime. (**c**) However, the laser operates in a distinct dynamic spatio-temporal regime, *I(t,T)*. The slow evolution time *T* is measured starting from an arbitrary moment in time. The intensity is colour-coded on a linear scale. Supplementary Movie 1 shows the pulse evolution.

As a test-bed system, we adopted a 1-km-long normal-dispersion ring-cavity fibre laser (see Fig. 1(a) and Supplementary Note 2 for details). Depending on the cavity parameters, the laser can operate either in the regime of high-quality mode-locking or in the regime of partial mode-locking^26^. We chose a relatively long cavity to have flexible access to a variety of complex partially mode-locked regimes. The laser generates a well-resolved pulse train with fairly stable inter-pulse separation (Fig. 1b). The pulse has an irregular envelope subject to substantial changes from pulse to pulse. In addition, pulses are filled in by smaller scale irregular noise-like structures (Fig. 1b). The experimental challenge here is to ascertain whether the laser is simply unstable and hops from one regime to another, or whether these radically differing temporal profiles belong to the same pulse breathing in the cavity under the influence of some complex nonlinear processes, leading to strong cyclic pulse re-shaping.

To answer this critical question, we experimentally characterized the laser's spatio-temporal intensity dynamics, *I(t,T)*, instead of analysing the usual one-dimensional intensity dynamics, *I(t)*; in other words, we measured how the instantaneous intensity pattern *I(t)* evolves over many-cavity round-trips, *T* (Fig. 1c). We did this using a technique that has been recently introduced and used in various systems^13^,^27^,^28^,^29^ (see Supplementary Note 3 for details of the technique).

The spatio-temporal representation of the laser's dynamics immediately reveals internal regularity and complex dynamics over a slow evolution coordinate *T*, of thought-to-be stochastic irregular radiation (Fig. 1c). We found that, despite the irregular time dynamics, the laser operates in a distinct spatio-temporal regime with pronounced deterministic evolution of the pulse's temporal envelope over slow evolution time *T*. Note the dynamic nature of the operational regime: the temporal profile of the pulse is strongly re-shaped from one round-trip to another. Although the spatio-temporal regime has a breathing structure, it is stable over a large number of round-trips. In this sense, the dynamical spatio-temporal regime is stationary; that is, the laser does not hop from one regime to another.

**Figure 2:**
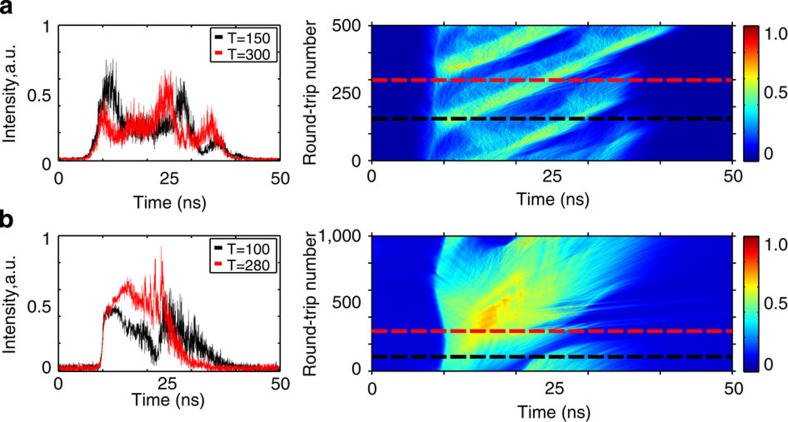
Periodicity in dynamical spatio-temporal regimes. Dynamical spatio-temporal regimes that are different in their periodicity properties are observed. Stochastic pulses could be (**a**) periodic and (**b**) localized, both over fast time *t* and slow evolution coordinate *T*. The right column demonstrates the spatio-temporal intensity dynamics, *I(t,T)*. The left-hand column shows the corresponding pulse profiles measured at evolution time *T* indicated with dotted lines. See corresponding Supplementary Movies 2 and 3.

Further, by adjusting the polarization controller or the pump power, we operate the laser in a set of dynamical spatio-temporal regimes that differ in their periodicity properties over slow evolution time *T* (Fig. 2). Regimes vary from one featuring completely homogenous evolution over round-trips (Supplementary Fig. 3) to spatio-temporal regimes with pronounced evolution periodicity (Fig. 2a) and, finally, to those in which the pulse tends to be localized over both the spatial and evolution coordinates (as in Fig. 2b). In all cases, the temporal shape of the pulse is subject to pronounced modification in the course of its evolution. The experimentally demonstrated periodicity and the existence of a long time scale over a slow evolution time, corresponding to hundreds of cavity round-trips, is completely unexpected. Our measurements clearly demonstrate that lasers exhibiting highly nonlinear dynamics are operated in distinct dynamical spatio-temporal regimes rather than in simple temporal regimes.

**Figure 3:**
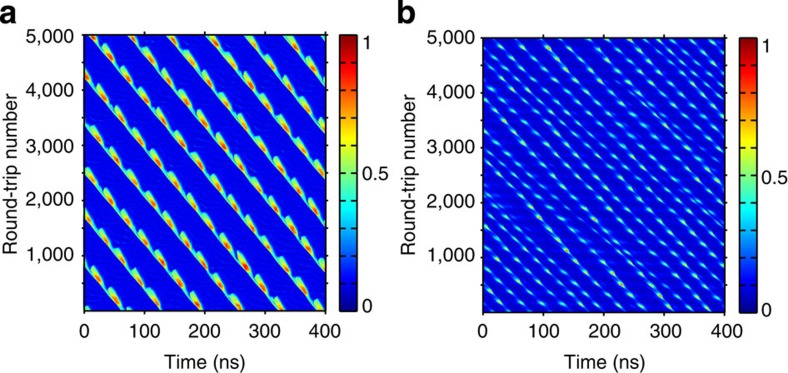
Long-term periodicity and internal interactions of stochastic pulses in dynamical spatio-temporal regimes. (**a**) Stable periodic (both over time and over evolution coordinate) train of stochastic pulses. (**b**) Interaction of localized stochastic pulses. See Supplementary Movies 4 and 5.

We then uncover non-trivial dynamics and stability over a large number of cavity round-trips. The dynamical spatio-temporal regime of stochastic pulses localized over both the fast time and slow evolution coordinate (Fig. 2b) is stable over a substantial observation time, Fig. 3a. Note the considerably different evolution time scales. The typical time scale of fluctuations within the pulse is only hundreds of picoseconds. The typical pulse width is tens of nanoseconds. However, the pulses are slowly evolved over a considerably different time scale of ∼400 round-trips (∼2 ms in terms of evolution time; Fig. 3a).

The laser can exhibit correlation in radiation on even larger time scales. We operated the laser in a dynamical spatio-temporal regime of a 10-ns-wide 100 round-trip-long localized stochastic, which interacts similar to coherent structures with typical interaction time of the order of 1,000 round-trips (Fig. 3b). The interaction manifests itself as scattering of one stochastic pulse upon another. In these scattering events, energy can be transferred from one pulse to another, leading to emergence of new pulses. The physical origin of the existence of multi-scales in spatio-temporal dynamics structures is still to be understood.

### Revealing localized structures via ACF's evolution mapping

Besides the existence of substantially different time scales, dynamic spatio-temporal regimes could contain various types of localized light structures embedded in stochastic radiation. To reveal the constituents of the radiation, we utilize an autocorrelation analysis. Later in the paper, we analyse the spatio-temporal regime presented in Fig. 1. We measure the intensity autocorrelation function (ACF) directly with a real-time oscilloscope to gain access to large de-tuning times *τ*, Fig. 3a. The zero-order ACF peak has two time scales: a narrow autocorrelation peak (of about 100-ps wide), which sits on top of a wider background (about 10-ns wide) (Fig. 3b). Each scale of the zero-order ACF peak appears from some temporal structures within the total radiation. In our case, the narrow ACF peak corresponds to the typical time scale of intensity fluctuations defining the stochastic nature of the pulse, while the broad pedestal reflects the average width of the noise-like pulse.

The zero-order ACF peak cannot distinguish between various temporal structures that have similar temporal width; they would be mixed up within the same temporal scale of the zero-order ACF peak. However, different types of structures may also have different group velocities and, consequently, round-trip times. Difference in round-trip time could be detected via analysis of higher-order ACF peaks: structures will manifest themselves as sub-peaks superimposed on the *N*-th order ACF peak and shifted over its centre (Fig. 3b).

Here, we introduce the ACF evolution map by plotting the N-th order ACF peak as the N-th layer in a two-dimensional plot, *K*(*τ*_1_,*N*) (Fig. 3c; see Supplementary Note 4 for details). Any temporal structure with a constant group velocity that is not equal to the speed of the co-moving reference frame will give a straight slanted line on the ACF evolution map, meaning that structures can be easily detected.

The ACF evolution map presents the novel technique of detection of structures travelling with various speeds and provides a lot of information. The number of slanted lines provides the number of different types of the structures embedded in the radiation. The length of each line corresponds to the typical lifetime of the quasi-stationary structure over a slow evolution time (cavity round-trips). By measuring the angle of each satellite line, the group velocity of each structure may be determined to precision as high as 10^−6^. The high precision value of group velocity can be further used to plot the spatio-temporal intensity dynamics *I(t, T)* in such reference frame, in which selected structures becomes immobile and thus directly visible and detectable. In particular, the presence of different structures of similar temporal width means that the autocorrelation function in its zero-order actually has multiple overlapping scales rather than two scales.

**Figure 4:**
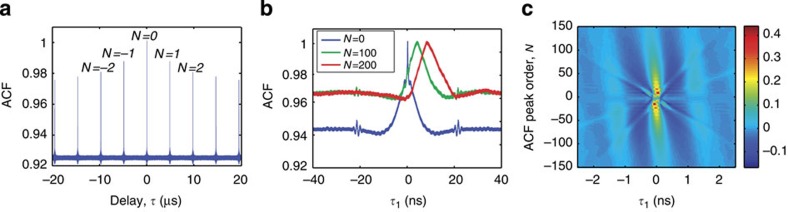
Intensity autocorrelation function's evolution mapping. (**a**) Intensity ACF, *K*(*τ*)=〈*I*(*t*)·*I*(*t*+*τ*)〉_*t*_, measured over large temporal de-tuning values. Peak separation equals the average cavity round-trip time, and *N*=0, ±1, ±2 and so on. denotes the order of the ACF peak. (**b**) Higher-order peaks of intensity ACF (normalized to unity). Time de-tuning *τ*_1_ is defined as *τ*_1_=*τ*−*N* × *T*_RT_. (**c**) The ACF evolution map, *K*(*τ*_1_,*N*), makes it possible to reveal radiation constituents. Each slanted line corresponds to some quasi-stationary structure. Colour is used to show the amount of autocorrelation.

Further, we use the ACF evolution map to explore distinct parts of radiation and reveal embedded localized structures. We found that, when analysed in a proper co-moving reference frame, a noise-like inter-pulse background exhibits a prominent spatio-temporal structure (see insert at Fig. 4a), despite it not revealing any visible structure, either in intensity dynamics or in initial spatio-temporal representation. This spatio-temporal structure corresponds to the laminar state recently observed in Raman fibre lasers^27^. Note that the group velocities of the stochastic pulse and the background differ by only 4 × 10^−5^. The laminar, coherent nature of the inter-pulse background can be confirmed by the background level of its ACF, which is close to unity, and also the prominent bell shape of its intensity probability distribution function (Supplementary Fig. 6). Thus, we have experimentally observed a generation of stochastic pulses sitting on the laminar coherent background; that is, the laser exhibits radically different coherent properties within the same dynamical spatio-temporal regime.

The laminar inter-pulse background is populated with a number of localized (over time) and quasi-stationary (over evolution time) dark structures (see insert at Fig. 4a). Similar dark traces were observed in the Raman fibre laser operating in the laminar regime and have been proved to be dark and grey solitons^27^. Dark and grey solitons can be also generated in partially mode-locked fibre lasers on the initial stage of the radiation build-up, as has been numerically shown in paper^30^. The ACF evolution mapping technique enables us to experimentally observe them directly for what we believe to be the first time in passively mode-locked fibre lasers.

**Figure 5:**
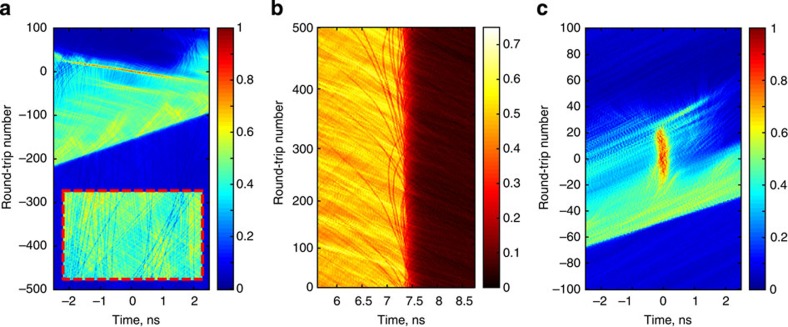
Co-existence and interaction of quasi-stationary localized structures with a stochastic pulse. The same dynamic spatio-temporal regime is plotted in different co-moving reference frames, the speeds of which are defined via ACF evolution mapping. (**a**) Laminar inter-pulse background with dark/grey solitons. A different colour code is used on the insert to make the structures visible. (**b**) Interaction of solitons with stochastic pulse. (**c**) Generation of bright localized structure within a stochastic pulse. Coordinate values in **b** and **c** correspond to those on **a**.

Further, by applying the ACF evolution mapping new technique, we have uncovered the intriguing dynamics of interaction of dark/grey solitons with the main pulse (Fig. 5b). There are different scenarios of interaction depending on the velocity difference between localized structures and the main pulse. Some dark solitons, after hitting the kink-type edge of the pulse, enter the pulse and become trapped inside, acquiring the pulse's speed, and eventually leave the pulse through its other edge, assuming a speed almost equal to the initial one (Fig. 5a). Other solitons ‘bounce' back from the pulse as demonstrated in Fig. 5b. This effect resembles full internal reflection in geometrical optics.

Finally, we observe the simultaneous generation of stochastic bursts of light, which freely propagate through the large-scale pulse envelope at a speed different to that of the pulse (Fig. 5a). In a reference frame moving with the pulse, such events look like high-intensity extreme walls (see Supplementary Fig. 8). In the reference frame co-moving with bright structures, we can localize these structures and estimate their lifetime over the slow evolution time (Fig. 5c). These stochastic bursts of light persist for up to 40 round-trips over the evolution coordinate. They can be generated in a quasi-periodic manner as well (Supplementary Fig. 9). Coherence properties of observed bright localized structures are still to be uncovered, as phase is not measured in our experiments.

## Discussion

The demonstrated dynamic spatio-temporal regimes in the generation of passively mode-locked fibre lasers delivering stochastic pulses reveal new possibilities for experimental studies of extremely complex and the diverse nonlinear physics behind their operation. Visibly stochastic generation regimes can now be experimentally classified on the basis of their spatio-temporal properties rather than just their temporal profiles. The proposed technique of ACF evolution mapping could be applied to a variety of cyclic fibre systems and lasers when nonlinearity leads to the emergence of coherent structures embedded into stochastic radiation; for example, through modulation instability triggered by noise or when coherent structures are generated in chaotic laser cavities. Note that coherent structures embedded in random radiation have been observed in other laser systems, for instance, breather-like structures emerging through modulation instability initiated by a broadband noise^17^ and the coherent structures in chaotic laser cavities^31^,^32^ and other systems^33^. In our work, we experimentally demonstrate co-existence and interaction of random and coherent radiation components in dynamic spatio-temporal regimes in fibre lasers.

Experimental observations of dynamical spatio-temporal regimes and localized structures within them bring new perspectives to many theoretical and numerical studies. Indeed, most of theoretical efforts to describe mode-locked lasers are currently focused on asymptotic regimes in which stable mode-locked pulses are observed. Not-so-stable regimes, which are far more numerous and richer in physics, are not usually considered because experimental confirmations were not feasible before now. The combination of numerical and experimental studies of new types of multi-scale dynamics in spatio-temporal regimes that are neither coherent (as in traditional mode-locked lasers) nor completely stochastic could result in a better understanding of the nonlinear physics behind the operation of practical devices.

We believe that the demonstrated approaches will bring about new methods and techniques that will help to uncover complex nonlinear intra-cavity dynamics. For instance, real-time measurements of spectrally resolved spatio-temporal dynamics will associate specific spatio-temporal patterns with distinct spectral features. Synchronous measurements of the spatio-temporal dynamics with real-time spectra by means of dispersion Fourier transform^34^,^35^ could help in the understanding of the origin of spectral fluctuations in real lasers. Triggering spatio-temporal dynamics measurements with the moment of time at which the pump wave is coupled into the system paves the way for experimental studies of mode correlations build-up from the initial noise at the start of mode-locked operation.

In the broader physics context, the existence of stochastic pulses propagating over a laminar (stable) background makes partially mode-locked fibre lasers very similar to classical hydrodynamic pipe systems with turbulent puffs propagating within the laminar fluid flow^36^. Thus, classical questions about how critical parameters govern the onset and decay of turbulence can also be posed in fibre laser experiments using partially mode-locked lasers.

We anticipate that experimental studies of dynamical spatio-temporal dynamics in various types of systems (not limited to cavity- and fibre-based ones) will lead to a deeper understanding of underlying complex nonlinear processes and may trigger the development of new types of engineering devices. More generally, any light source exhibiting complex intermittent temporal dynamics may also have hidden long-term correlations that can be now experimentally studied and understood.

## Additional information

**How to cite this article**: Churkin, D.V. *et al*. Stochasticity, periodicity and localized light structures in partially mode-locked fibre lasers. *Nat. Commun*. 6:7004 doi: 10.1038/ncomms8004 (2015).

## Supplementary Material

Supplementary Information Supplementary Figures 1-9, Supplementary Notes 1-5 and Supplementary References.

Supplementary Movie 1 shows synchronous temporal and spatio-temporal evolution in the generation regime from Fig. 1.

Supplementary Movie 2 shows synchronous temporal and spatio-temporal evolution in the generation regime from Fig. 2a.

Supplementary Movie 3 shows synchronous temporal and spatio-temporal evolution in the generation regime from Fig. 2b.

Supplementary Movie 4 shows synchronous temporal and spatio-temporal evolution in the generation regime from Fig. 3a.

Supplementary Movie 5 shows synchronous temporal and spatio-temporal evolution in the generation regime from Fig. 3b.
