# Supplementary material for: Stochasticity, periodicity and localized light structures in partially mode-locked fibre lasers
Source: Nat Commun. 2015 May 7;6:7004. doi: 10.1038/ncomms8004 (PMC4430819; doi:10.1038/ncomms8004)
Supplement: Supplementary Information — Supplementary Figures 1-9, Supplementary Notes 1-5 and Supplementary References. [file ncomms8004-s1.pdf]

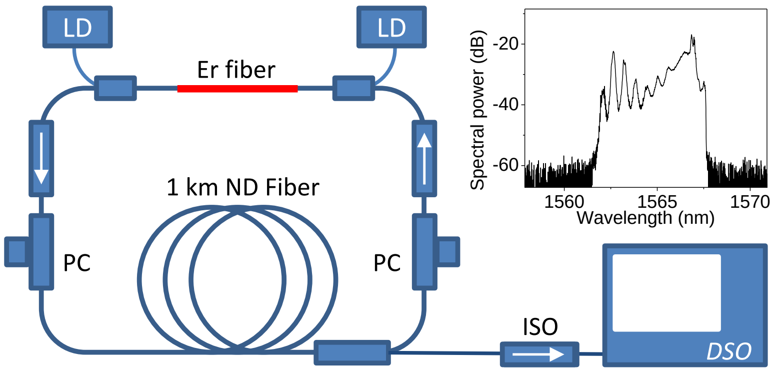

Supplementary Figure 1. Schematic representation of the experimental set up. Er-DC – Erbium-doped fibre gain medium, LD – laser diode (pump source for Er-DC), PC1, PC2 – polarisation controllers, PBS – polarisation beam splitter, ND – 1-km normal-dispersion fibre ($D = -44$ ps/nm/km), DSO – Digital storage oscilloscope. *Inset* – typical optical spectra observed.

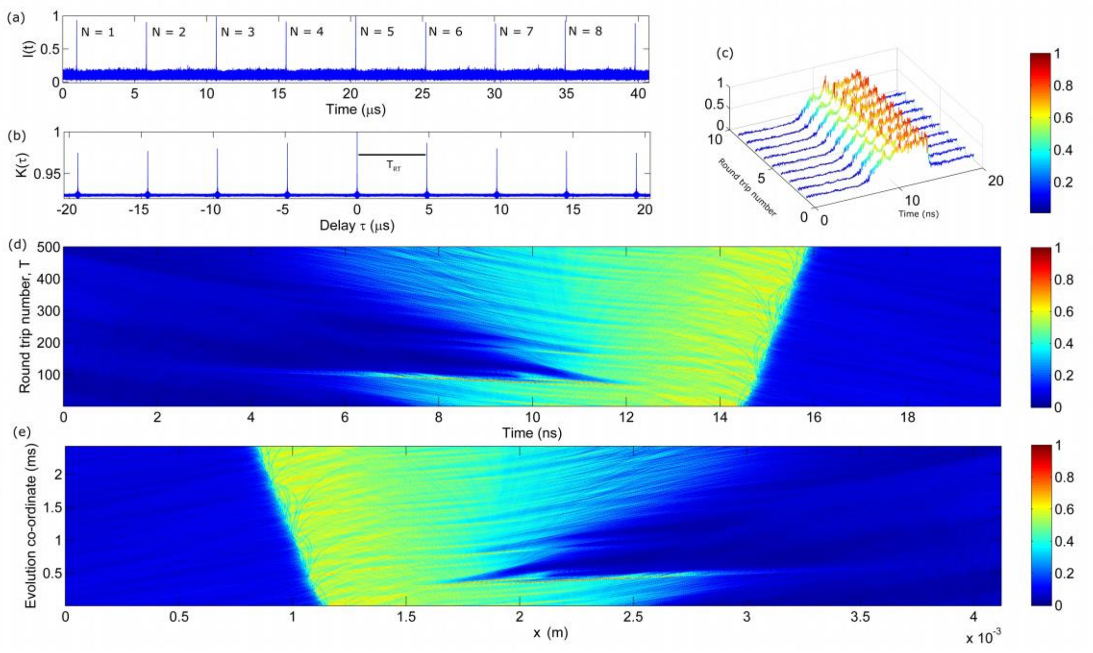

Supplementary Figure 2. Principle of real-time measurement of spatio-temporal intensity dynamics. (a) The intensity dynamics $I(t)$ of the laser. (b) The autocorrelation function $K(\tau)$ exhibits well pronounced peaks at the round trip period T_{RT}. (c) The obtained period length can then be used to precisely select samples of the time series located round trips apart and arranged row-wise. Proceeding over the whole data set $I(t)$ in this fashion, one then arrives at the spatio-temporal dynamics (d). The equivalent spatio-temporal dynamics representation is given in (e), which shows how spatial distribution of intensity along the fibre evolves over slow evolution time.

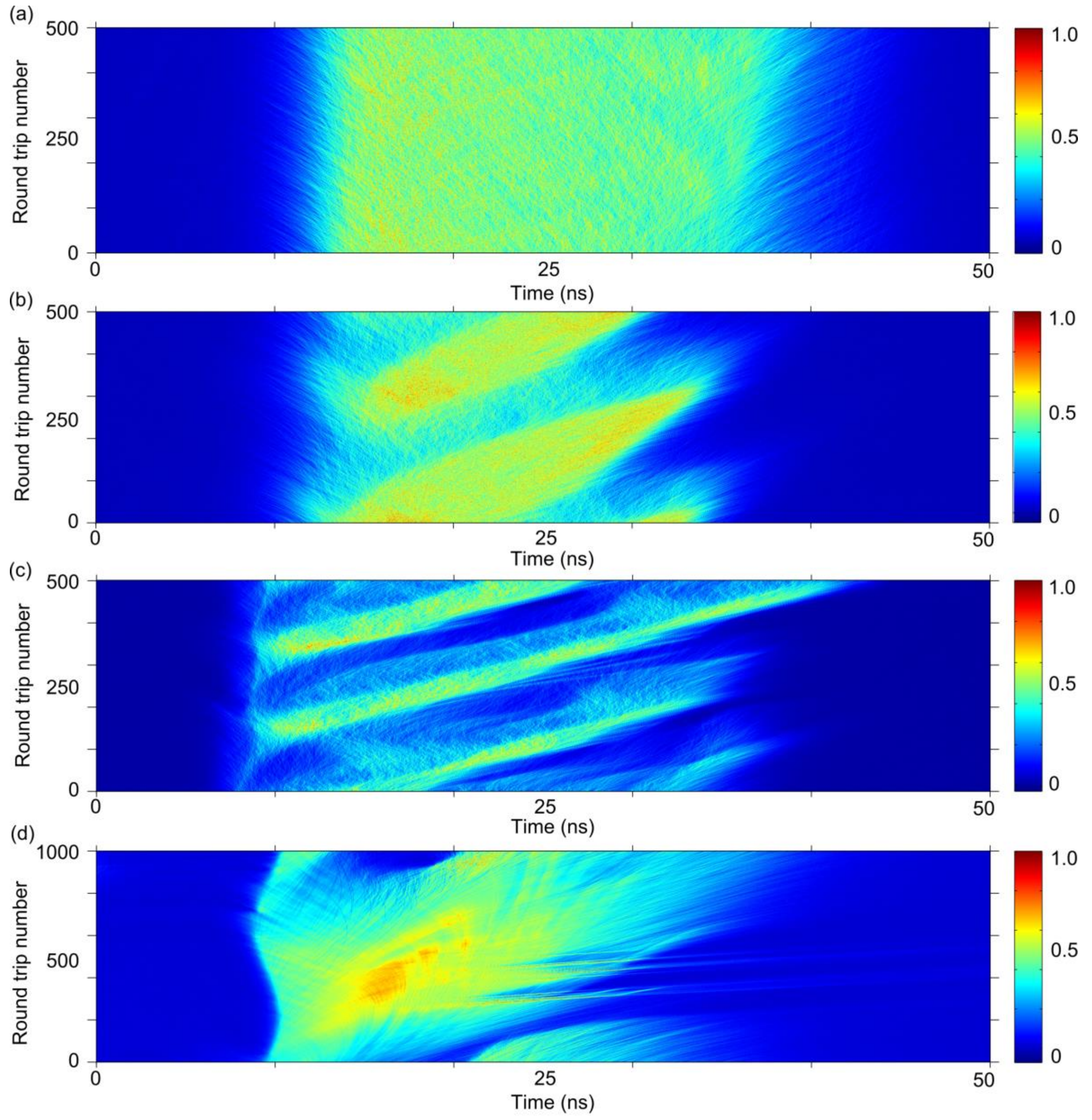

Supplementary Figure 3. Different dynamic spatio-temporal regimes of long passively mode-locked fibre laser delivering stochastic pulses.

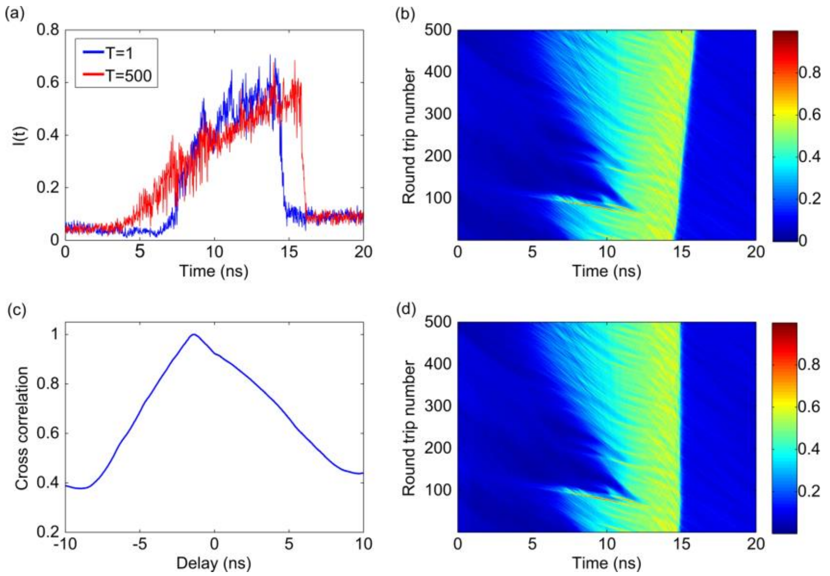

Supplementary Figure 4. Correction of resolution-related skew in spatio-temporal dynamics. The skew results from limited accuracy of round-trip time measurement due to finite discretisation time of the oscilloscope and accumulates over consecutive round trips. (a) Time dynamics in a co-moving reference frame shifted after 500 round-trips due to uncertainty in the round-trip time value. (b) Initial uncorrected spatio-temporal dynamics. (c) Cross-correlation function is used to obtain the shift accumulated over a given number of round trips. The resolution-related error appears as a shift of the peak from the location of zero delay. The value of the shift, in its turn, can be used in an affine transformation routine to de-skew the spatio-temporal dynamics. (d) Corrected spatio-temporal dynamics. The pulse front now is completely immobile in the corresponding co-moving reference frame.

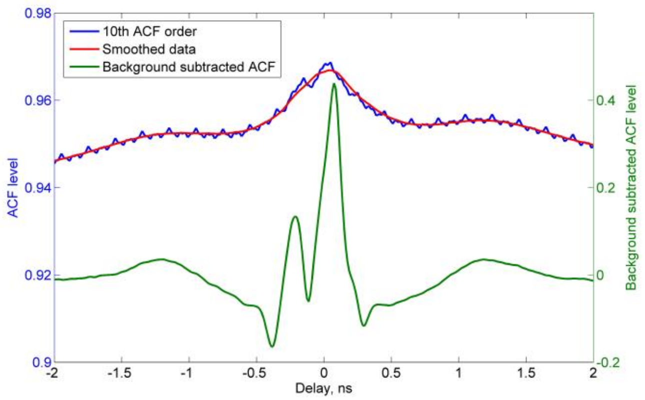

Supplementary Figure 5. Subtraction procedure to reveal small amplitude auto-correlation peaks. Blue curve – initial intensity ACF for the 10-th order ACF peak. Red curve – averaged auto-correlation trace. Green curve – the averaged and normalized result of subtraction of the average auto-correlation trace from the initial auto-correlation trace.

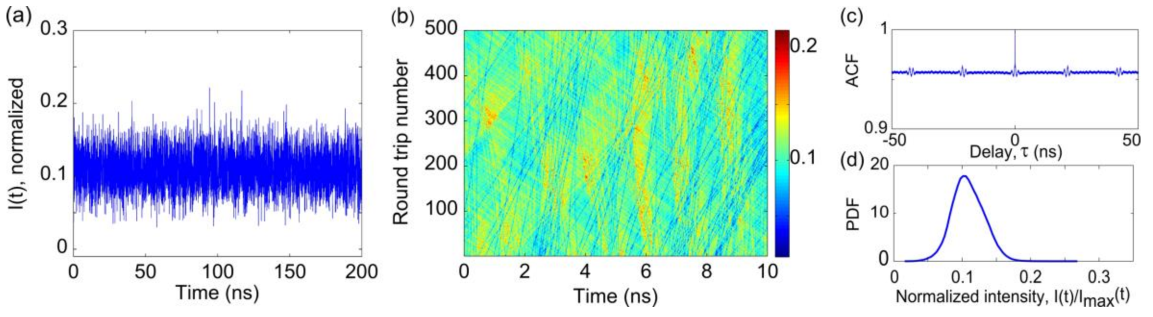

Supplementary Figure 6. Intensity dynamics of the inter-pulse laminar background. (a). Typical one-dimensional time trace of inter-pulse background dynamics observed in partially mode locked lasers. (b) Spatio-temporal intensity dynamics of the inter-pulse radiation. Laminar inter-pulse background is coherent with suppressed intensity fluctuations. The background is furrowed by numerous dark and grey solitons (blue curved traces) living on it. (c) Intensity autocorrelation function and (d) intensity probability density function measured for the inter-pulse background only (excluding main pulses). The intensity is normalised to the peak value occurring in the regime.

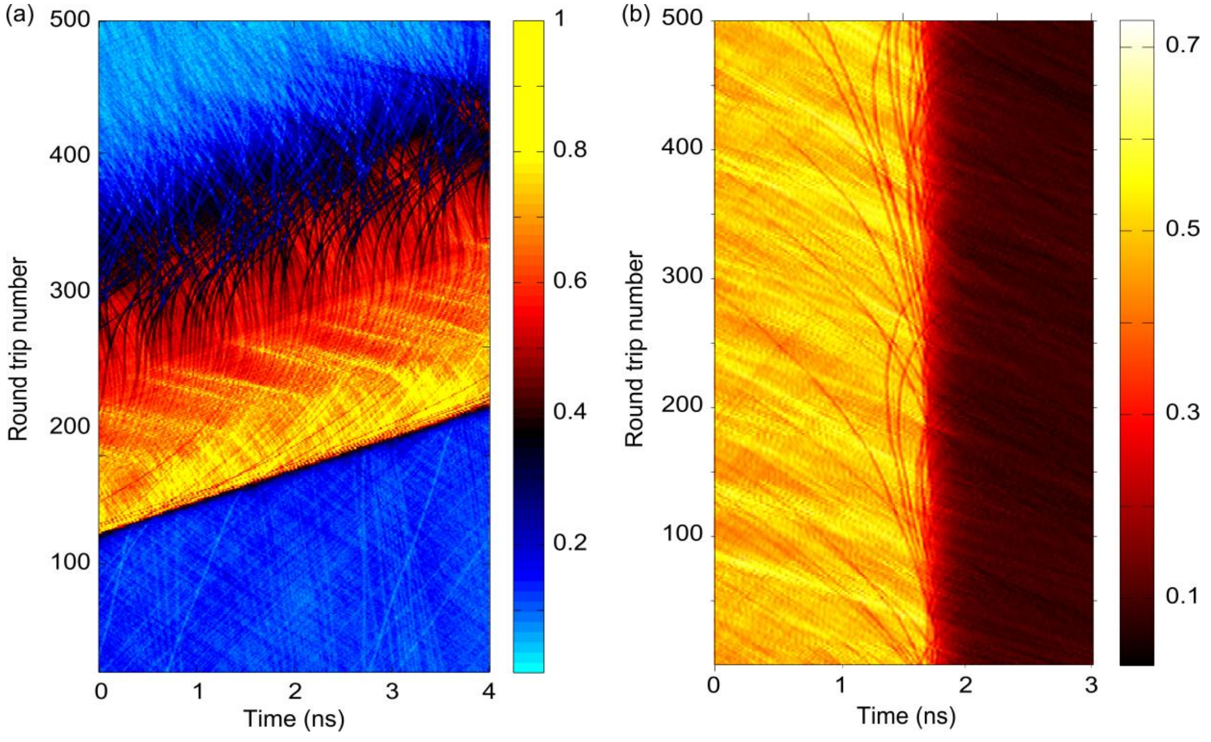

Supplementary Figure 7. Co-existence and interaction of dark and bright localized structures with a stochastic pulse. (a, b) Interaction of dark and grey solitons with a stochastic pulse. Different color codes are used on (a) and (b) to highlight the dark/grey solitons propagating from the laminar background and leaving the stochastic pulse again.

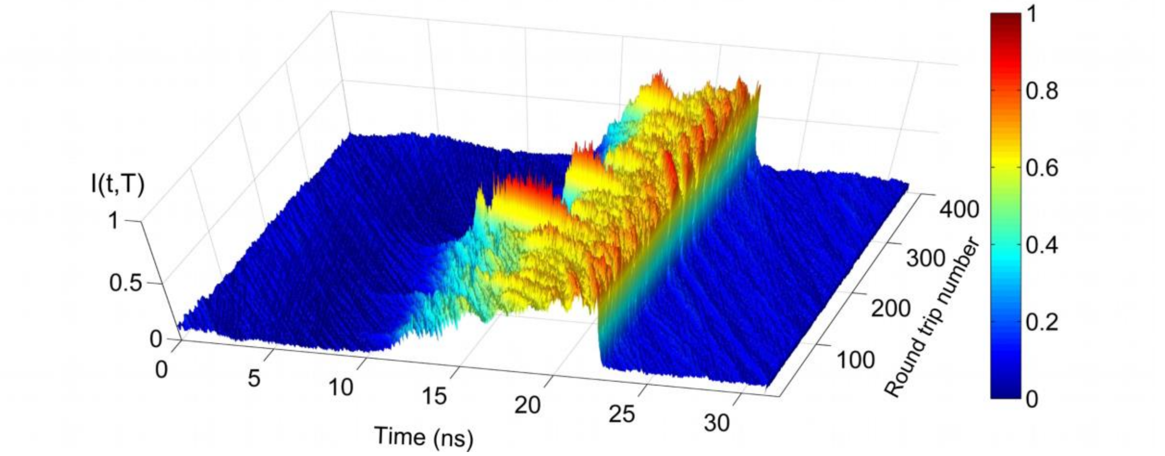

Supplementary Figure 8. 3D representation of experimentally measured dynamic spatio-temporal regime. Emergence of a bright localized structure in the form of a vertical wall is clearly visible. The same dynamic spatio-temporal regime as in Fig. 1 of the main text is shown here.

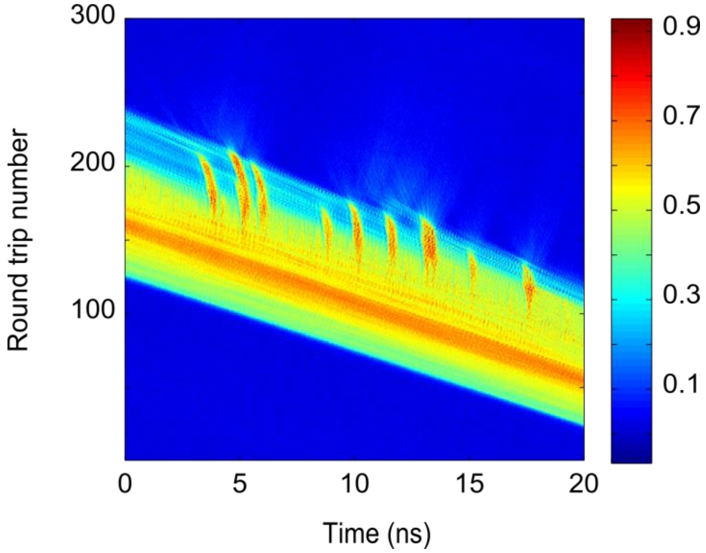

Supplementary Figure 9. Slow moving spatio-temporally localized bright structures which are genrerated quasi-periodically. The spatio-temporal regime from Supplementary Figure 2d is shown.

Supplementary Note 1. Definitions

We define the following quantities which will be used further on in the text:

a. Mean value of the laser intensity.

Let $\boldsymbol{I(t)}$ denote the time-dependent output intensity of the laser, where $\boldsymbol{t}$ is the instantaneous time instant. Then the mean intensity value is written as

$$\overline{I(t)} = \frac{1}{\Delta\Gamma}\int_{-\Delta\Gamma/2}^{\Delta\Gamma/2} I(t)\cdot dt,$$

where $\Delta\Gamma$ is a suitably chosen time interval; and

b. Autocorrelation function (ACF) of the laser intensity

$$K(\tau) \equiv \langle I(t) \cdot I(t+\tau) \rangle$$
$$= \int_{-\infty}^{\infty} I(t) \cdot I^*(t+\tau) \cdot dt$$
$$= \int_{-\infty}^{\infty} I(t) \cdot I(t+\tau) \cdot dt$$

It must be noted that the above quantities are defined for a continuous variable $I(t)$. However, the real signal $I(t)$ is discrete because of digital sampling, and the appropriate discrete forms of the above estimators are used:

$$\overline{\mathbf{I(t)}} = \frac{\mathbf{1}}{\mathbf{N}} \sum_{i=1}^{N} \mathbf{I(t)}\,;$$

$$K(\tau) = \begin{cases} \dfrac{1}{N-|\tau|} \cdot \displaystyle\sum_{\tau=0}^{N-\tau+1} I(t) \cdot I(t+\tau)\,,\ 0 \le \tau \le N, \\ \dfrac{1}{N-|\tau|} \cdot K^*(\tau),\ -N \le \tau < 0. \end{cases}$$

Here the discrete sum is divided by the factor $N - |\tau|$ to remove the bias arising from the finite length of the time series.

Supplementary Note 2. Experimental setup

We study dynamic spatio-temporal generation regimes of the following NPE-mode-locked long fibre laser, Supplementary Figure 1, in which both complete and partial mode locking could be triggered due to nonlinear polarization evolution (NPE). In order to trigger mode locking in these lasers, polarisation controllers are used that allow conversion of input light of arbitrary polarisation to output light of any desired polarization. As it is well known, complete transformation of the radiation polarisation state requires several phase retarders with at least three degrees of freedom. Large number of parameters for setting the radiation polarisation state in combination with laser cavity properties and adjustable pump power gives rise to a plethora of pulsed generation regimes including both regimes with complete mode locking and those with partial mode locking, these latter featuring much worse pulse parameter stability compared to the former. We designed a ring fibre laser in which various controller polarisation settings could be used to trigger both completely mode-locked (see recent reviews in the field [1, 2]) and partially mode-locked regimes, the latter differing from the former in the dynamics of their pulse parameter evolution from one cavity round trip to another. However, there is no defined boundary between these regime types and it is possible to trigger any of them with different settings of the polarisation controllers, producing pulses of different structure even under complete mode locking [1] and different dynamics as far as optical turbulence[2, 3].

The laser cavity included a stretch of Erbium-doped fibre as the gain medium and a 1-km normal dispersion fibre ($D = -44$ ps/nm/km). Typical average output power measured after the polarisation beam splitter PBS was of the order of 20 mW. The laser cavity round-trip time T_{RT} was approximately 5 μs.
A 25-GHz real time digital storage oscilloscope (DSO) together with a 50-GHz photodetector was used to record the long-term intensity dynamics. The DSO we used provides temporal resolution of 40 ps. The size of the inbuilt memory of the DSO puts an upper limit on the length of recordable time traces. The total trace length together with the sampling rate define the maximum number of round-trips over which the spatio-temporal regime can be captured in real-time. In our case, this was limited to a number between thousand round-trips up to hundreds of thousands round-trips depending on the chosen temporal resolution.

The laser could be operated in a number of regimes being different in their intensity dynamics [4]. In our experiments, different operation regimes were accessed by changing orientations of the polarisation

controllers and/or pump power. In this paper, we focus mostly on highly stochastic regimes with partial mode locking that exhibit so pronounced and complex non-linear dynamics that they are usually not considered for study. In particular, although the regime shown in Fig. 1 of the main text produces a regular pulse train, it also concentrates a very significant amount of energy (up to 90%) in the inter-pulse background, in contrast to the conventional regimes of completely mode-locked systems [5-8] where all the energy is contained in pulses. This results in complex processes of interaction of the stochastic pulse with the background.

Supplementary Note 3. Measurements of spatio-temporal intensity dynamics

Usually, the laser dynamics is measured as a one-dimensional time series, $\boldsymbol{I}(\boldsymbol{t})$. Here we describe in details real-time measurements of spatio-temporal intensity dynamics. Similar methodology has been used to interpret observations related to interaction between solitons in a driven cavity [9, 10] and to explain formation and interaction of vector dissipative solitons in external-cavity semiconductor lasers [11]. Note that in numerical modelling the spatio-temporal representation is widely used to uncover underlying physics behind of operation of different types of light sources, see, for example [12-22] . One can define two dimensional spatio-temporal dynamics, $\boldsymbol{I}(\boldsymbol{t},\boldsymbol{T})$, of the laser intensity evolution by slicing the time trace of the laser intensity $\boldsymbol{I}(\boldsymbol{t})$ into intervals equal to some time period $\boldsymbol{T}$, and then using them to form the rows of a matrix, Supplementary Figure 2. Here the instantaneous fast time co-ordinate $\boldsymbol{t}$ may be mapped (because the fastest of the involved processes is linear propagation with the speed of light *c*) onto the real space co-ordinate along the fibre, $\boldsymbol{x}$, via transformation $\boldsymbol{t}-\boldsymbol{x}/\boldsymbol{c}$, while slow evolution time $\boldsymbol{T}$ is measured in terms of number of cavity round-trips. Alternatively, the period $\boldsymbol{T}$ can be any internal period of complex non-linear time dynamics over which evolution periodicity is expected due to any specific considerations. In the case of cavity-based systems, it is natural to choose the time period *T* equal to the cavity round-trip time $\boldsymbol{T}_{\mathbf{RT}}$.

Supplementary Figure 2 illustrates the process of spatio-temporal dynamics measurement on an example of a spatio-temporal regime presented in Fig. 1 of the main text. Supplementary Figure 2a shows the intensity dynamics of the laser. Autocorrelation function $\boldsymbol{K}(\boldsymbol{\tau})$ of the output (Supplementary Figure 2b) exhibits well pronounced peaks at intervals corresponding to the internal period in the system, in our case equal to the round trip time $\boldsymbol{T}_{\mathbf{RT}}$. This value of $\boldsymbol{T}_{\mathbf{RT}}$ can now be used to select smaller sections of the time trace from the original time series $\boldsymbol{I}(\boldsymbol{t})$ (Supplementary Figure 2a), which are spaced a round trip time period apart. These sections can then be arranged in the form of a matrix $\boldsymbol{I}(\boldsymbol{t},\boldsymbol{T})$, where each row now corresponds to a particular round trip number (Supplementary Figure 2c). Continuing in this fashion over a large number of round trips, one arrives at the spatio-temporal intensity dynamics as depicted in Supplementary Figure 2d and in the main text. The described procedure is a generalisation of the stroboscopic-type measurements previously used, for example, in experimental characterisation of soliton rains in fibre lasers [23, 24]. Note that the described procedure is physically equivalent to choosing some reference frame co-moving at such speed $\boldsymbol{v}_{\mathbf{g}}$ that the reference frame covers a cavity of length *L* in round-trip time $\boldsymbol{T}_{\mathbf{RT}}$ exactly, $\boldsymbol{v}_{\mathbf{g}}=\boldsymbol{L}/\boldsymbol{T}_{\mathbf{RT}}$. In such a co-moving reference frame, the intensity pattern of interest within spatio-temporal intensity dynamics $\boldsymbol{I}(\boldsymbol{t},\boldsymbol{T})$ remains stationary, *i.e.* does not move. Further, in this co-moving reference frame, the evolution coordinate initially defined in terms of round-trips physically corresponds to the slow evolution time measured as $\boldsymbol{T}=\boldsymbol{N}\cdot\boldsymbol{T}_{\mathbf{RT}}$, over which one observes the slow evolution of the chosen intensity pattern. Here $\boldsymbol{N}$ is the number of round-trips completed by this pattern. At the same time, the intensity pattern measured over instantaneous time $\boldsymbol{t}$ corresponds to the spatial distribution of the intensity along the fibre by the transform $\boldsymbol{t}-\boldsymbol{L}/\boldsymbol{v}_{\mathbf{g}}$. In this sense, we can consider the measured intensity dynamics as spatio-temporal intensity dynamics $\boldsymbol{I}(\boldsymbol{x},\boldsymbol{T})$, Supplementary Figure 2e. Note the change of the sign of the horizontal axis in Supplementary Figure 2e compared to Supplementary Figure 2d. In practice, however, it is more convenient to keep the spatio-temporal intensity dynamics in the initial variables $\boldsymbol{t}$ and $\boldsymbol{T}$ (measured in number of round-trips) just to establish direct equivalence with commonly studied intensity dynamics $\boldsymbol{I}(\boldsymbol{t})$ measured, actually, in a stationary reference frame. We use the representation of dynamics in variables $(\boldsymbol{t},\boldsymbol{T})$ everywhere henceforward, as well as throughout the main text. Note that the oscilloscope records temporal

events in order of occurrence, hence features arriving earlier along the time axis in Supplementary Figure 2d correspond to the leading edge of the pulse.

Supplementary Figure S3 is a high-resolution reproduction of the spatio-temporal dynamics presented in Figure 2 of the main text. The pulses which were assumed to be of chaotic nature clearly show well-defined periodicity over slow evolution time at different scales. Furthermore, these features survive and maintain their shape over hundreds of round trips, or equivalently many non-linear/dispersion lengths, thereby revealing their coherent nature. It is interesting to note how spatio-temporal dynamics allows direct detection of short-lived localized structures like dark/grey solitons (specifically in Supplementary Figure 3d), which would have otherwise remained indistinguishable from noise in one-dimensional intensity dynamics.

In our experiments, the value of the period used in setting the speed of the co-moving reference frame, in which the spatio-temporal dynamics is measured, can be obtained in a straightforward manner by using an RF spectrum analyser. Alternatively, the same information can be extracted from the time domain by calculating autocorrelation $\boldsymbol{K(\tau)}$ of $\boldsymbol{I(t)}$, which exhibits periodical peaks at intervals equal to round trip time $\boldsymbol{T_{\mathrm{RT}}}$. The round trip time can be estimated to the accuracy of $\boldsymbol{\delta t/T_{\mathrm{RT}}}$, corresponding in frequency domain to expression

$$\Delta\nu_{RT} = \frac{1}{{T_{RT}}^2}\delta t,$$

where $\boldsymbol{\delta t}$ is temporal resolution of the DSO. The factor $\boldsymbol{\delta t/T_{\mathrm{RT}} \approx 2.5 \times 10^{-6}}$ in the present case of a 1-km cavity, so frequency accuracy is approximately 4 Hz. This is very close to the resolution of state-of-the-art RF analysers. Nevertheless, despite the ratio of the oscilloscope discretisation time to the round-trip time being in our case as low as 2×10^{-6}, this is not enough to define the speed of the co-moving reference frame with accuracy sufficient to immobilise the feature of interest. As a result, the measured spatio-temporal dynamics will exhibit a skew, which cannot be corrected by a single-pixel shift of the round trip, see Supplementary Figure 2a as an example where the pulse front is slanted. While this does not affect qualitative interpretation of data, such skews could potentially introduce errors when making quantitative estimates along the slow-evolution co-ordinate $\boldsymbol{T}$.

To correct for this, a simple shear image transformation can be used. A shear operation is an affine transformation of the image matrix, which is represented by the pointwise operation:

$$\begin{bmatrix} x' \\ y' \end{bmatrix} = \begin{bmatrix} 1 & a \\ 0 & 1 \end{bmatrix} \begin{bmatrix} x \\ y \end{bmatrix},$$

i.e., $\boldsymbol{x' = x + ay}$

$\boldsymbol{y' = y}$

Essentially this means that while the $\boldsymbol{y}$ co-ordinates of the image are preserved, the $\boldsymbol{x}$ co-ordinates are translated. Such a co-ordinate transformation requires remapping of the transformed values to a regular grid, which in turn requires interpolation. In our case, we need to find the value of $\boldsymbol{a}$, which is the error in the measured round trip value. To do that, two rows $\boldsymbol{p}$ and $\boldsymbol{q}$ spaced by an appropriate number of round trips are chosen from the matrix $\boldsymbol{I(t,T)}$. Cross-correlation between these rows would produce a correlation peak shifted away from the zero-delay location (Supplementary Figure 4c). The value of the shift gives a measure of the accumulated error over $\boldsymbol{(p - q + 1)}$ round trips. The value of $\boldsymbol{a}$ is then the error per round trip, which can be obtained by dividing the obtained shift by the number of round trips $\boldsymbol{(p - q + 1)}$. Using this value of $\boldsymbol{a}$ in the affine transform, we can reconstruct the deskewed ST dynamics as shown in Supplementary Figure 4d.

Supplementary Note 4. Isolation of radiation constituents via evolution mapping of intensity autocorrelation function

Spatio-temporal dynamics reveals the existence of long-lived localized structures within the pulse, which move along different inclinations with respect to the vertical, Figure S3. These inclinations arise due to differences between the velocity v_{g} of the co-moving reference frame and that of the localized structures. Localized structures become directly visible on spatio-temporal intensity dynamics if an appropriate reference frame in which they are immobile is used. To define the speed of such a frame we define with high precision the

corresponding round-trip time of different parts of the radiation with a precision up to several picoseconds. To do that, we use higher-order autocorrelation analysis. We measure the intensity autocorrelation function (ACF) $(\tau) = \langle I(t) \cdot I(t+\tau) \rangle_t$. Note that usually ACF is measured by intensity auto-correlator, so the range of detuning time τ is limited to hundreds of picoseconds. In addition, an auto-correlator provides data averaged over many pulses, so only average pulse properties could be restored. Here we measure ACF directly with a real-time oscilloscope over substantially large time (up to milliseconds) to have access to large detuning times τ. In such measurements, single-shot aurocorrelation of particular pulse can be calculated. Obviously, the temporal resolution of ACF is limited in this case by the oscilloscope bandwidth. It was around 40 ps in our case, while sub-ps resolution can be achieved easily for average ACF measured by an auto-correlator. In our experiments, we measure the autocorrelation function over a sufficiently long record of intensity dynamics $I(t)$. In particular, the *N*-th order ACF peak appears as a result of convolution of the intensity pattern with its replica shifted by N cavity round-trips (*i.e.* the same intensity pattern measured N cavity round-trips later). The zeroeth-order ACF peak results from two overlaid pulse train replicas appears at $\tau = 0$ and has two time scales: a narrow autocorrelation peak (of about 100-ps width) sitting on top of a wider background (about 10 ns wide) (see Fig. 3b). In our case, the narrow ACF peak corresponds to the typical time scale of intensity fluctuations defining the stochastic nature of the pulse, while the broad pedestal reflects the average width of the noise-like pulse. Note that although we observe a double-scale ACF, it is different from the one previously studied in mode-locked lasers[1, 4]. Indeed, in our case of partial mode locking, stochastic pulses are much broader, so the smaller scale in the zero-order ACF for broad pulses is of similar temporal width as the wide pedestal of more stable pulses in the aforementioned papers.

As discussed in the main text, the relative difference between the group velocities of the underlying features result in a relative shift of the sub peaks with respect to the broad pulse, and also between each other. As we measure the autocorrelation function over a sufficiently long record of intensity dynamics $I(t)$ (*i.e.* much longer than the round-trip time), even small differences between the round trip periodicities accumulate and thus become resolvable. Similar to time domain, one-dimensional representation of intensity ACF does not allow us to identify different structures rigorously. This is first of all caused by extremely small typical group velocity differences between different types of localized structures and dispersive waves which could be as small as 10^{-4}–10^{-5}, see Ref. [27]. Another obstacle is a small amount of energy in those structures compared to the total energy in the radiation leading to tiny, poorly resolved features in the *N*-th order ACF peak, Fig. 3b, and making any quantitative analysis impossible. To overcome this problem, we build a two-dimensional intensity ACF evolution map by plotting the ACF signal over time detuning τ_1 and number N. Here $N = 0, \pm 1, \pm 2$, *etc.* is the order of the ACF peak effectively representing the number of round-trips and time detuning $\tau_1 = \tau - N \cdot T_{\mathrm{RT}}$ so that $|\tau_1| < T_{\mathrm{RT}}/2$.

To improve the visibility of the sub-peaks further, we can remove the contribution of this broad part by calculation of a moving average of the initial auto-correlation trace, Supplementary Figure 5 (red curve), and subtract the result from the initial auto-correlation trace. The resulting auto-correlation function, Supplementary Figure 5 (green curve), also smoothed with a moving average filter, comprises a number of highly visible peaks, which may be associated with different structures. We can further repeat these steps for different orders of auto-correlation peaks and plot the result in the form of an evolution map of auto-correlation functions shown on Fig. 3c of the main text. As a result of this procedure, a number of trajectories with different slopes are clearly visible. Each trajectory arises from a particular type of structures (or a family of structures) within the spatio-temporal intensity dynamics, which move at the same group velocity; the slope of these trajectories on the ACF evolution map is directly proportional to their group velocities. The ACF evolution map of Fig. 3c thus provides a systematic way of identification and differentiation between various types of complex radiation constituents. Using precise values of the group velocity obtained from ACF evolution map, one can plot spatio-temporal intensity dynamics in corresponding co-moving reference frame to highlight the structures of interest.

Supplementary Note 5. The laminar inter-pulse background and pulse-background interactions

To further explore distinct parts of radiation propagating at different velocities, we use various values of group velocities obtained from ACF evolution map. First, we take the group velocity value differing by $\mathbf{4 \cdot 10^{-5}}$ from the initial value. We find that this corresponds to the inter-pulse background, which has low, but non-zero intensity and is supposed to contain pure noise as indicated by simple intensity dynamics, Supplementary Figure 6a. The presence of inter-pulse background is one of the unique features of partially mode-locked regimes, whereas even operating in noise-like/double-scale lasing regimes the laser may produce background-free pulse train[1], in which the pulse-to-pulse period approaches the upper state lifetime of the active medium. Quite to the contrary, in partially mode-locking regimes, the spatio-temporal dynamics of the inter-pulse background plotted in an appropriate co-moving reference frame exhibits a prominent structure, Supplementary Figure 6b, corresponding to the laminar state recently observed in Raman fibre lasers [25].

Spatio-temporal representation of laser dynamics allows us to study temporal properties of the radiation separately for different clusters of radiation and to gain better understanding of their internal structure. For example, here we plot the spatio-temporal dynamics for the inter-pulse background and study intensity ACF and probability density function (PDF) measured for the inter-pulse background only, Supplementary Figure 4c,d. The ACF level is close to unity and PDF has a prominent bell-shaped peak that proves the coherent nature of the background. Existence of stochastic pulses propagating over a laminar background makes partially mode-locked fibre lasers very similar to classical hydrodynamic pipe systems with turbulent puffs propagating within the laminar fluid flow [26]. Thus, classical questions on how critical parameters govern the turbulence onset and decay can be also posed in fibre-laser experiments using partially mode-locked lasers.

Note dark traces on the inter-pulse background in Supplementary Figure 6b. Each trace has a typical length of hundreds of non-linear lengths. It is known that dark and grey solitons may emerge on a condensate (laminar) background and be stable there [27]. Dark and grey solitons also proved to be generated on a condensate background in laminar regime of quasi-CW Raman fibre lasers [25]. Kelleher et a l[21] recently showed numerically that dark/grey solitons are generated at the initial stage of radiation build-up in passively mode-locked fibre lasers, although they eventually decay.

The background carries dark and grey solitons, which propagate at a group velocity different from the main nanosecond-wide bright pulse. Furthermore, they interact with the main pulse in different ways, as discussed in the main text. Here we provide high-resolution figures, which are a reproduction of Fig. 5a,b of the main text, and which highlight the details of such interaction, Supplementary Figure 7. Supplementary Figure 8 (also compare with Fig. 5c of main text) shows the emergence of a bright quasi-stationary localized structure in the reference frame of the main pulse. In this reference frame, the localized structure looks like sheer walls of light on top of the stochastic pulse.

Supplementary References

1. S. Smirnov, S. Kobtsev, S. Kukarin, and A. Ivanenko, "Three key regimes of single pulse generation per round trip of all-normal-dispersion fiber lasers mode-locked with nonlinear polarization rotation," Optics Express **20**, 27447-27453 (2012).
2. F. Kärtner, D. Zumbühl, and N. Matuschek, "Turbulence in mode-locked lasers," Physical review letters **82**, 4428 (1999).
3. S. Wabnitz, "Optical turbulence in fiber lasers," Optics letters **39**, 1362-1365 (2014).
4. S. Kobtsev, S. Smirnov, S. Kukarin, and S. Turitsyn, "Mode-locked fiber lasers with significant variability of generation regimes," Optical Fiber Technology (2014).
5. A. Chong, W. H. Renninger, and F. W. Wise, "All-normal-dispersion femtosecond fiber laser with pulse energy above 20nJ," Optics letters **32**, 2408-2410 (2007).
6. F. W. Wise, A. Chong, and W. H. Renninger, "High-energy femtosecond fiber lasers based on pulse propagation at normal dispersion," Laser and Photonics Reviews **2**, 58-73 (2008).

7. S. Kobtsev, S. Kukarin, and Y. Fedotov, "Ultra-low repetition rate mode-locked fiber laser with high-energy pulses," Optics express **16**, 21936-21941 (2008).
8. S. Kobtsev, S. Kukarin, S. Smirnov, S. Turitsyn, and A. Latkin, "Generation of double-scale femto/pico-secondoptical lumps in mode-locked fiber lasers," Optics express **17**, 20707-20713 (2009).
9. F. Leo, S. Coen, P. Kockaert, S.-P. Gorza, P. Emplit, and M. Haelterman, "Temporal cavity solitons in one-dimensional Kerr media as bits in an all-optical buffer," Nature Photonics **4**, 471-476 (2010).
10. J. K. Jang, M. Erkintalo, S. G. Murdoch, and S. Coen, "Ultraweak long-range interactions of solitons observed over astronomical distances," Nature Photonics **7**, 657-663 (2013).
11. M. Marconi, J. Javaloyes, S. Barland, S. Balle, and M. Giudici, "Vectorial dissipative solitons in vertical-cavity surface-emitting Lasers with delays," arXiv preprint arXiv:1406.0730 (2014).
12. J. M. Dudley, G. Genty, and S. Coen, "Supercontinuum generation in photonic crystal fiber," Reviews of modern physics **78**, 1135 (2006).
13. A. Zaviyalov, R. Iliew, O. Egorov, and F. Lederer, "Multi-soliton complexes in mode-locked fiber lasers," Applied Physics B **104**, 513-521 (2011).
14. D. Korobko, R. Gumenyuk, I. Zolotovskii, and O. Okhotnikov, "Multisoliton complexes in fiber lasers," Optical Fiber Technology (2014).
15. L. Zhao and D. Tang, "Generation of 15-nJ bunched noise-like pulses with 93-nm bandwidth in an erbium-doped fiber ring laser," Applied Physics B **83**, 553-557 (2006).
16. M. Erkintalo, Y. Xu, S. Murdoch, J. Dudley, and G. Genty, "Cascaded phase matching and nonlinear symmetry breaking in fiber frequency combs," Physical review letters **109**, 223904 (2012).
17. P. Grelu and J. Soto-Crespo, "Temporal Soliton “Molecules” in Mode-Locked Lasers: Collisions, Pulsations, and Vibrations," in *Dissipative solitons: from optics to biology and medicine* (Springer, 2008), pp. 1-37.
18. F. Leo, L. Gelens, P. Emplit, M. Haelterman, and S. Coen, "Dynamics of one-dimensional Kerr cavity solitons," Optics express **21**, 9180-9191 (2013).
19. A. Yulin, R. Driben, B. Malomed, and D. Skryabin, "Soliton interaction mediated by cascaded four wave mixing with dispersive waves," Optics express **21**, 14481-14486 (2013).
20. M. Schmidberger, D. Novoa, F. Biancalana, P. S. J. Russell, and N. Joly, "Multistability and spontaneous breaking in pulse-shape symmetry in fiber ring cavities," Optics express **22**, 3045-3053 (2014).
21. E. Kelleher and J. Travers, "Chirped pulse formation dynamics in ultra-long mode-locked fiber lasers," Optics letters **39**, 1398-1401 (2014).
22. A. Demircan, S. Amiranashvili, C. Brée, C. Mahnke, F. Mitschke, and G. Steinmeyer, "Rogue events in the group velocity horizon," Scientific reports **2**(2012).
23. S. Chouli and P. Grelu, "Rains of solitons in a fiber laser," Optics express **17**, 11776-11781 (2009).
24. S. Chouli and P. Grelu, "Soliton rains in a fiber laser: An experimental study," Physical Review A **81**, 063829 (2010).
25. E. G. Turitsyna, S. V. Smirnov, S. Sugavanam, N. Tarasov, X. Shu, S. A. Babin, E. V. Podivilov, D. V. Churkin, G. Falkovich, and S. K. Turitsyn, "The laminar-turbulent transition in a fibre laser," Nature Photonics **7**, 783-786 (2013).
26. K. Avila, D. Moxey, A. de Lozar, M. Avila, D. Barkley, and B. Hof, "The onset of turbulence in pipe flow," Science **333**, 192-196 (2011).
27. Y. S. Kivshar and B. Luther-Davies, "Dark optical solitons: physics and applications," Physics Reports **298**, 81-197 (1998).
